# Rumination in Children with Social Anxiety Disorder: Effects of Cognitive Distraction and Relation to Social Stress Processing

**DOI:** 10.1007/s10802-021-00837-6

**Published:** 2021-06-18

**Authors:** Leonie Rabea Lidle, Julian Schmitz

**Affiliations:** 1 Institute of Psychology, Department for Clinical Child and Adolescent Psychology, Leipzig University, Neumarkt 9-19, 04109 Leipzig, Germany; 2 Leipzig Research Centre for Early Child Development, Leipzig University, Leipzig, Germany

**Keywords:** Anticipatory processing, Post-event processing, Rumination, Social anxiety disorder, Children, Distraction

## Abstract

According to cognitive models of social anxiety disorder (SAD), both anticipatory processing and post-event processing are core mechanisms in disorder maintenance leading to dysfunctional coping with social situations through negative self-evaluation and increased anxiety. To date, little is known about these processes during late childhood, a critical period for disorder development. Further, it remains unclear if dysfunctional rumination in children can be altered through psychotherapeutic interventions such as cognitive distraction. In the current study, children aged 9 to 13 years with SAD and age- and gender-matched healthy controls (HCs, each: *n* = 30) participated in an experimental laboratory social stress task while anticipatory processing, post-event processing, subjective anxiety, self-evaluations, and autonomic arousal (skin conductance level) were assessed. Further, the impact of a brief cognitive distraction intervention on post-event processing was assessed. Children with SAD reported more negative anticipatory and post-event processing compared to HC children. Further, negative anticipatory processing was associated with higher subjective anxiety and reduced subjective performance ratings during the social stress task. In the aftermath of the stressor, distraction led to reduced subjective anxiety in the group with SAD and lower autonomic arousal in all children but did not alter post-event processing. The current study suggests that both anticipatory and post-event processing already play a key role in the maintenance of SAD in childhood. While distraction may be beneficial in reducing prolonged subjective anxiety and autonomic arousal after social situations, more research on interventions targeting ruminative processes is needed.

Social anxiety disorder (SAD), characterized by a persistent fear of negative evaluation, is one of the most prevalent anxiety disorders in childhood and youth (Beesdo et al., 2009). SAD typically emerges early in life, with the average age of onset in late childhood to midteens (Kessler et al., 2005). Commonly diagnosed among clinically anxious children seeking help for anxiety (e.g., Waite & Creswell, 2014), SAD is a significant burden for health care systems. Yet, children and adolescents with SAD are less likely to respond favorably to cognitive-behavioral therapy (CBT) when compared to adults (e.g., Halldorsson & Creswell, 2017), highlighting the importance of studying cognitive maintenance processes in childhood SAD to improve psychotherapy effectiveness.

## Rumination in Cognitive Models of SAD

Cognitive models of adult SAD propose that ruminative processes play an important role in the maintenance of the disorder (Clark & Wells, 1995; Hofmann, 2007). In detail, Clark and Wells (1995) differentiated two ruminative processes: (a) anticipatory processing occurring prior to social situations and (b) post-event processing occurring in the aftermath. During anticipatory processing, individuals dwell on negative outcomes of the impending social situation (e.g., “everybody will see how scared I am”), recall past social failures, and generate negative mental images (Clark & Wells, 1995). Anticipatory processing is thought to lead to a negatively biased processing of social situations, including increased anticipatory anxiety and expectations of poor performance (Clark & Wells, 1995). Post-event processing, on the other hand, is defined as a repetitive and detailed review of subjective negative experiences following a social situation (Clark & Wells, 1995). The individual engages in a “postmortem” (Clark & Wells, 1995), focusing on negative aspects of the past situation and retrieval of other past social failures, such as “I always embarrass myself in social interactions” (Modini & Abbott, 2016).

In accordance with cognitive models, several recent reviews concluded that anticipatory and post-event processing are key cognitive mechanisms in the maintenance of social anxiety in *adulthood* (Modini & Abbott, 2016; Penney & Abbott, 2014; Wong, 2016). However, as both anticipatory and post-event processing require complex cognitive processes and self-referential thinking, which develop in mid to late childhood (Alfano et al., 2002), the validity of these cognitive models for children remains uncertain. In this vein, several studies demonstrated that in late childhood between the ages of 8 and 13 years, cognitive processes, and in particular negative post-event processing (Schmitz et al., 2010), gain importance in the development and maintenance of SAD (for a review, see Halldorsson & Creswell, 2017).

## Anticipatory and Post-event Processing in Childhood SAD

To date, only a few studies have investigated negative anticipatory processing in children. Some studies found an association between childhood social anxiety and more negative anticipatory self-evaluations regarding upcoming social situations (e.g., Morgan & Banerjee, 2006; Tuschen-Caffier et al., 2011; for an exception see Halldorsson et al., 2019). Furthermore, negative anticipatory processing was shown to be associated with higher levels of social anxiety in a community sample aged 11 to 14 years (Hodson et al., 2008). Very few studies investigated anticipatory processing in nonclinical samples of children using experimental designs (Vassilopoulos et al., 2014, 2017). For instance, Vassilopoulos et al. (2014) instructed a community sample of children aged 10 to 11 years to engage in either guided anticipatory processing or distraction after announcing a public reading task. Relative to distraction, negative anticipatory processing led to maintained subjective anxiety, negative self-evaluations, and more catastrophic thinking. This effect was more pronounced in children with higher levels of social anxiety.

More studies have been done on post-event processing in children with SAD. A higher frequency of negative post-event processing was shown in both subclinical (Hodson et al., 2008; Schmitz et al., 2011) and clinical (Asbrand, Schmitz, et al., 2019b; Schmitz et al., 2010) samples with SAD compared to non-socially anxious controls. Negative post-event processing in socially anxious children seems to persist up to 1 week (Asbrand, Schmitz, et al., 2019b; Schmitz et al., 2011) and seems to lead to decreased self-evaluations over time (Schmitz et al., 2011). Negative post-event processing is associated with social anxiety even when depressive symptoms are statistically controlled (Schmitz et al., 2010), which is important given that depression is commonly associated with negative rumination (Thomsen, 2006). Further, negative post-event processing was identified as a risk factor for increased avoidance of social situations across adolescence (Miers et al., 2014), indicating that it might not only be a maintenance factor but may play a role in the development of SAD.

Since mental health in (older) children seems to be characterized by fewer negative but not more positive cognitions (Kendall & Chansky, 1991), positive anticipatory and post-event processing have been studied to a lesser extent. For example, Vassilopoulos et al. (2017) did not find an association between positive anticipatory processing and social anxiety in a community sample aged 12 to 13 years. Also, studies on positive post-event processing have been inconclusive: While one study reported less positive post-event processing in a sample with SAD aged 8 to 12 years compared to nonanxious controls (Schmitz et al., 2010), other studies did not find reduced levels of positive post-event processing in highly socially anxious children aged 10 to 12 years (Schmitz et al., 2011) and a clinical sample with SAD aged 9 to 13 years (Asbrand, Schmitz, et al., 2019b).

In summary, previous research suggests that childhood SAD may be associated with negative cognitive processing before and after social stress. But several research questions remain unanswered. Regarding *anticipatory processing*, clinical samples of children with SAD remain understudied so it remains unclear if findings from community samples (e.g., Vassilopoulos et al., 2014) generalize to clinical populations. Also, as most studies regarding anticipatory processing employed retrospective questionnaire-based designs (e.g., Hodson et al., 2008) and mostly assessed anticipatory self-evaluations but not negative anticipatory processing (e.g., Morgan & Banerjee, 2006), more experimental research is needed to prospectively evaluate anticipatory processing and its proposed negative consequences (e.g., elevated anxiety levels; see Clark & Wells, 1995), under well-controlled conditions. In this context, social evaluative speech tasks have been successfully implemented to induce ruminative processes in samples with SAD (e.g., Schmitz et al., 2011).

Regarding *post-event processing*, research on therapeutic interventions specifically targeting negative post-event processing is so far lacking in clinically socially anxious children. This is particularly troublesome as children with SAD profit less from existing CBT programs than children with other anxiety disorders (Hudson et al., 2015), and because negative post-event processing was shown to be insufficiently addressed in a group-based CBT in children aged 9 to 13 years (Asbrand, Schmitz, et al., 2019b). A promising strategy to reduce negative post-event processing in adults is cognitive distraction. Distraction refers to diverting attention away from recurrent negative thoughts and turning it to neutral or pleasant thoughts or actions (Roelofs et al., 2009). Studies evaluating the benefits of distraction compared to guided negative rumination in highly socially anxious undergraduates demonstrated that distraction was associated with less negative post-event processing (Blackie & Kocovski, 2016), more positive post-event processing (Kocovski et al., 2011), and a decrease in subjective anxiety (Wong & Moulds, 2009). In an adult SAD sample, Rowa et al. (2014) reported a positive effect of distraction compared to focusing on the performance on an impromptu speech task on anxiety, but a negative effect on negative post-event processing. However, these findings might be attributable to baseline differences in symptom severity and anxiety (Rowa et al., 2014). In childhood SAD effects of cognitive distraction on post-event processing remain understudied.

Last, most studies in children have measured anticipatory and post-event processing mainly by assessing subjective measures of stress processing. Since behavioral measures and self-reports of arousal do not always converge in child and youth samples (Miers et al., 2011), psychophysiological measures such as skin conductance level (SCL), which is considered a reliable indicator of perceived threat (Lovibond, 1992), can provide distinct information about stress-related autonomic responding beyond what can be collected from self-reports (Los Reyes et al., 2012).

## The Current Study

Taking the limitations of previous studies as a starting point, in the current study we aimed to assess both anticipatory and post-event processing during a well-controlled experimental social stress task in a clinical sample of children with SAD and a healthy control (HC) group. We further aimed to measure the effects of a cognitive distraction intervention implemented directly after the social stress task on post-event processing while assessing subjective and autonomic stress markers. We postulated the following hypotheses:

### Hypothesis 1.

In anticipation of a social evaluative situation, children with SAD will report (a) more negative anticipatory processing, (b) comparable positive anticipatory processing, and (c) higher levels of subjective anxiety, and will (d) show higher autonomic arousal compared to a HC group (Clark & Wells, 1995; Vassilopoulos et al., 2014).

### Hypothesis 2.

Negative anticipatory processing will be associated with (a) higher subjective anxiety during the social evaluative situation and (b) lower subjective performance ratings across all participants (Vassilopoulos et al., 2014).

### Hypothesis 3.

 After a social evaluative situation, children with SAD will report (a) more negative post-event processing and (b) comparable positive post-event processing (Schmitz et al., 2010, 2011).

### Hypothesis 4.

 After a social evaluative situation, cognitive distraction will lead to (a) less negative post-event processing, (b) comparable positive post-event processing, (c) reduced subjective anxiety, and (d) lower levels of autonomic arousal (Blackie & Kocovski, 2016; Rowa et al., 2014). This effect will be more pronounced in children with SAD than in HCs.

## Method

### Experimental Design

The study employed a 2 × 2 × 2 mixed repeated measures design, consisting of two experimental groups (SAD vs. HC), two experimental conditions (distraction vs. uninstructed post-event processing) and two repeated measurement points (Stress Task 1 [T1], Stress Task 2 [T2]). All participants took part in both experimental conditions. The order was counterbalanced within each group.

### Participants

Children aged 9 to 13 years were recruited through information letters, flyers, and advertisements. Following a telephone screening, eligible families were invited to take the Kinder-DIPS (Margraf et al., 2017), a modified German version of the Anxiety Disorders Interview Schedule for Children–Revised (Silverman & Nelles, 1988). Inclusion criterion for the group with SAD was a primary diagnosis of SAD according to the fifth edition of the *Diagnostic and Statistical Manual of Mental Disorders (DSM-5*; American Psychiatric Association, 2013). Children in the HC group did not have any lifetime diagnosis of a mental disorder. Exclusion criteria for all children were health conditions that could alter psychophysiological assessment (e.g., asthma, cardiac arrhythmia, methylphenidate) and past or current psychological treatment. Initially, 62 participants were included in the study but two children with SAD had to be excluded due to irregularities in the study procedure. Participant characteristics are found in Table 1. All participants spoke German fluently, and only one participant in the HC group did not report German as their first language. The following comorbid disorders were present in the group with SAD: specific phobia (*n* = 11), general anxiety disorder (*n* = 6), child separation anxiety disorder (*n* = 2), elective mutism (*n* = 1), sleeping disorders (*n* = 1), depressive disorders (*n* = 1), and dyslexia (*n* = 1).

**Table 1:** Participant Characteristics

Characteristic	Group		Statistics
	Children with social anxiety disorder	Healthy controls	
Sample size (*n*)	30	30	
Mean age (*SD*), in years	11.6 (1.1)	11.6 (1.1)	*ns* ^a^
Female (%)	66.7	60.0	*ns* ^b^
Mean SASC-R (*SD*)	52.7 (13.0)	28.4 (6.2)	*p* < 0.001 ^a^
Mean CDI (*SD*)	17.8 (9.2)	5.9 (3.5)	*p* < 0.001 ^a^
Mean BMI (*SD*)	18.5 (2.8)	19.56 (3.6)	*ns* ^a^
School (%)			
Grammar school (%)	86.7	76.7	*ns* ^b^
Comprehensive school (%)	10.0	10.0	*ns* ^b^
Primary school (%)	3.33	6.67	*ns* ^b^
Other (%)	0.00	6.67	*ns* ^b^
Parental marital status (% separated)	40.0	3.33	*p* < 0.001 ^b^

*CDI* Children’s Depression Inventory, *SASC-R* Social Anxiety Scale—Revised, *BMI* Body mass index (kg/m^2^)

^a^Based on *t* test

^b^Based on chi-square test

### Ethical Considerations

This study was granted ethical approval by the University’s Research Ethics Committee. Parents and children were both provided with written and verbal information about the study. To participate in the study, written parental consent and child assent were both required. All participants received a child-appropriate voucher (30€) as compensation and children in the clinical group were offered treatment in the department’s outpatient clinic.

### Procedure

**Fig. 1:**
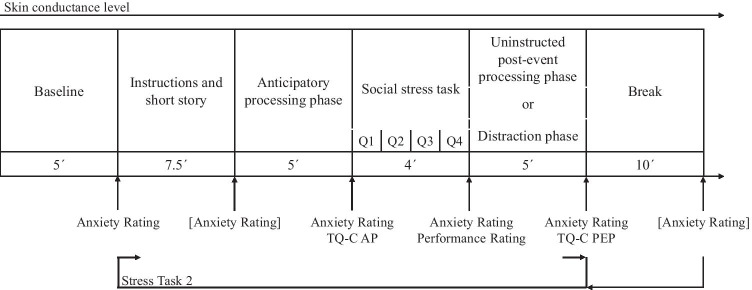
Experimental Procedure. *Note.* Overall procedure including the social stress task consisting of four standardized questions (Q1 to Q4); each participant completed the task twice. Measures not included in the analysis are shown in brackets. TQ-C-AP = Thoughts Questionnaire for Children—Anticipatory Processing; TQ-C-PEP = Thoughts Questionnaire for Children—Post-event Processing; Performance Rating = children’s retrospective self-rating of performance; Anxiety Rating = children’s retrospective self-rating of anxiety

After children arrived at the laboratory, psychophysiological measuring devices were attached and a 5-min baseline was taken. Next, all children participated in a social stress task consisting of a social performance situation during which the children had to answer questions regarding a short story in front of two unknown female observers (Schmitz et al., 2011, 2012). The children completed the task twice. Prior to each task, a 5-min uninstructed anticipatory processing phase was implemented during which children were told to wait while the experimenter checked the psychophysiological measures before the experiment could proceed. No further instructions were given, to allow participants to ruminate spontaneously. During the following social stress task, children answered four standardized questions posed by two female adult observers about a previously heard short story. Children had 1 min to hear and answer each question and were instructed to do their best, because the observers and peers would later rate their performance based on video recordings (Schmitz et al., 2011; Spence et al., 1999). All observers received a briefing and were trained to give standardized neutral verbal and nonverbal feedback while maintaining a friendly attitude. After the first social stress task, participants were randomly assigned to one of two conditions: In the uninstructed post-event processing condition, children waited 5 min for the experiment to proceed without further instructions, allowing for spontaneous post-event processing. In the distraction condition, participants played a noncompetitive memory game (Majong 3 Free.Ink, 1C Wireless, download: 14.06.2017) on a tablet. Tablet-based virtual games have been proven successful in inducing cognitive distraction (e.g., Hagenaars et al., 2017). After a 10-min break, the described sequence was repeated for the second implementation of the social stress task (see Fig. 1).

### Psychometric Measures

#### Structured Diagnostic Interview With Children and Parents

Children were assigned diagnoses based on the Kinder-DIPS (Margraf et al., 2017), which was separately administered to the child and a parent. It enables standardized clinical assessment of lifetime diagnoses, current and past, according to the criteria of the 10th revision of the *International Statistical Classification of Diseases and Related Health Problems* (*ICD-10*; World Health Organization, 2011) and the *DSM-5* (American Psychiatric Association, 2013). All interviewers were trained in the administration of the Kinder-DIPS. Diagnostic sessions were videotaped and continuously supervised; diagnoses were discussed with a consensus team led by an experienced clinical psychologist.

#### Social Anxiety Scale for Children—Revised

The Social Anxiety Scale for Children—Revised (La Greca & Stone, 1993) is a 22-item self-report questionnaire assessing social anxiety in children aged 8 to 16 years. Items can be grouped into two subscales measuring fear of negative evaluation (e.g., “I worry about what other kids think of me”) and social anxiety and distress (e.g., “I feel nervous when I talk to kids I don’t know very well”). Items are rated on a 5-point Likert scale ranging from 1 (*not at all*) to 5 (*all the time*). Total scores can range from 18 to 90. The internal consistency was satisfactory in a community sample aged 9 to 13 years (α = 0.78; La Greca & Stone, 1993) and excellent in the current sample (α = 0.95).

#### Children’s Depression Inventory

The Children’s Depression Inventory (CDI; Kovacs, 1985) is a self-report questionnaire measuring depressive symptoms according to the *DSM-5* in children aged 8 to 16 years. The German version (Stiensmeier-Pelster et al., 2014) consists of 29 items and total scores can range from 0 to 58. For each item, children are asked to choose one of three statements that best describes the way they have been feeling lately (e.g., “I am sad once in a while” [0], “I am sad many times” [1], or “I am sad all the time” [2]). The CDI differentiates between children aged 9–12 years with and without depression (sensitivity: 91.7%; specificity: 81.9%; Frühe et al., 2012) and has good internal consistency (α = 0.87; 8–16 years; Stiensmeier-Pelster et al., 2014). In our study the internal consistency was excellent (α = 0.94).

#### Thoughts Questionnaire for Children

The Thoughts Questionnaire for Children (Schmitz et al., 2010, 2011) is a self-report instrument measuring the frequency of post-event processing (“How often did you think…”) in the aftermath of a social stress task. It consists of 14 items, seven positive and seven negative cognitions, referring to the performance on the social stress task (e.g., “I did well on the task” [positive]), the observers (e.g., “The observers didn’t like me” [negative]), and the feelings experienced (e.g., “I felt anxious” [negative]). Children rate the frequency of each cognition on a 6-point Likert scale ranging from 0 (*never)* to 5 (*very often)*. To measure anticipatory cognition, a parallel version was built by rewording the items (Penney & Abbott, 2015) so that they referred to the upcoming social stress task (e.g., “I am going to feel anxious”). The internal consistency in our sample was good to excellent for negative (T1/2: α = 0.93/0.96) and positive (T1/2: α = 0.81/0.87) anticipatory processing and excellent for negative (T1/2: α = 0.96/0.97) and positive (T1/2: α = 0.91/0.93) post-event processing.

#### State Anxiety

Participants rated their anxiety levels at several points throughout the experimental procedure (e.g., “How scared were you while answering questions about the story in front of the two observers?”; see Fig. 1) on a visual analogue scale ranging from 0 (*no anxiety*) to 10 (*extreme anxiety*; Schmitz et al., 2010, 2011).

#### Subjective Performance Ratings

Immediately following each social stress task, participants rated their own performance on a 1 (*excellent*) to 6 (*insufficient*) scale corresponding to the German school grading system (Schmitz et al., 2010). Lower scores indicate higher performance.

### Psychophysiological Measures

Electrodermal activity was recorded with the VU-AMS Ambulatory Monitoring System (Geus et al., 1995; Willemsen et al., 1996). Two silver/silver chloride electrodes were attached to the medial phalanges of the index and middle finger of the participant’s nondominant hand. SCL was measured throughout the whole experiment and data were preprocessed using automatic artifact detection and low-pass filtering (for a detailed description, see http://www.vu-ams.nl/). Means of SCL relative to baseline were calculated for relevant periods (e.g., anticipatory processing phase).

### Data Analysis

Statistical analyses were conducted using the open statistics software R (R Core Team, 2018). Hypotheses were evaluated via mixed linear models (MLMs) to account for the hierarchical nature of the data. The mixed-models packages lme (Bates et al., 2014) and lmerTest (Kuznetsova et al., 2017) were used and the level of significance was set at α = 0.05 for all statistical analyses. As proposed by Luke (2017), models were fitted using restricted maximum likelihood and *p* values were derived with Kenward–Roger approximation to account for small sample sizes. To analyze Hypothesis 1a and b, the MLM was fitted with one between-subjects factor, Group (SAD, HC), and two within-subject factors, Scale (positive anticipatory processing, negative anticipatory processing) and Task (T1, T2), and all possible interaction terms as fixed effects. To analyze Hypothesis 1c and d, the MLM was fitted with one between-subjects factor, Group (SAD, HC), and one within-subject factor, Task (T1, T2), and all possible interactions as fixed effects. To analyze Hypotheses 3 and 4, corresponding MLMs were built, adding Condition (distraction, uninstructed post-event processing) as a third within-subject factor. All models included a random intercept to control for subject effects. To analyze Hypothesis 2, stepwise multiple regression models were conducted. To analyze Hypothesis 2a, trait social anxiety, and negative and positive anticipatory processing were included as predictors of subjective anxiety during the social stress tasks. Because of possible developmental influences (Alfano et al., 2002) and group differences in depression scores, age as well as depression scores were included as control variables. To analyze Hypothesis 2b, the regression model additionally included state anxiety levels as predictor of subjective performance ratings as these differed significantly between groups during the social stress tasks (see Manipulation Check). Averaged values of both social stress tasks were used for the analyses. Adjusted *R*^2^, the Akaike information criterion, and significant increases in predictive validity are reported for each regression model. An a priori power analysis (Faul et al., 2007) indicated a sample size of *n* = 27 participants per group based on a medium effect size (Schmitz et al., 2010) and a power of (1 – β) = 0.95.

## Results

### Manipulation Check

Children with SAD and the HC group did not differ in their subjective anxiety scores assessed directly after the 5-min baseline, *t*(58) = -1.41, *p* = 0.164, *d* = 0.36*,* 95% confidence interval (CI) [-0.16, 0.89]. The manipulation check revealed a robust increase in subjective anxiety from baseline to the social stress tasks in both groups at both tasks, group with SAD: all *t*s ≥ 9.24, all *p*s < 0.001, all *d*s ≥ 1.94; HC group: all *t*s ≥ 3.98, all *p*s < 0.001, all *d*s ≥ 0.70. Children with SAD reported higher subjective anxiety than the HC group during T1 and T2, all *t*s ≥ 6.85, all *p*s < 0.001, all *d*s ≥ 1.77. There was a significant increase in SCL between baseline and the stress task in both groups in T1 and T2, all *t*s ≥ 5.25, *p*s < 0.001, *d*s ≥ 0.54, but no difference in SCL between groups during T1 and T2, all *t*s < 0.80, *p*s > 0.428.

### Anticipatory Processing

#### Hypothesis 1a and b: Frequency of Negative and Positive Anticipatory Processing

**Fig. 2:**
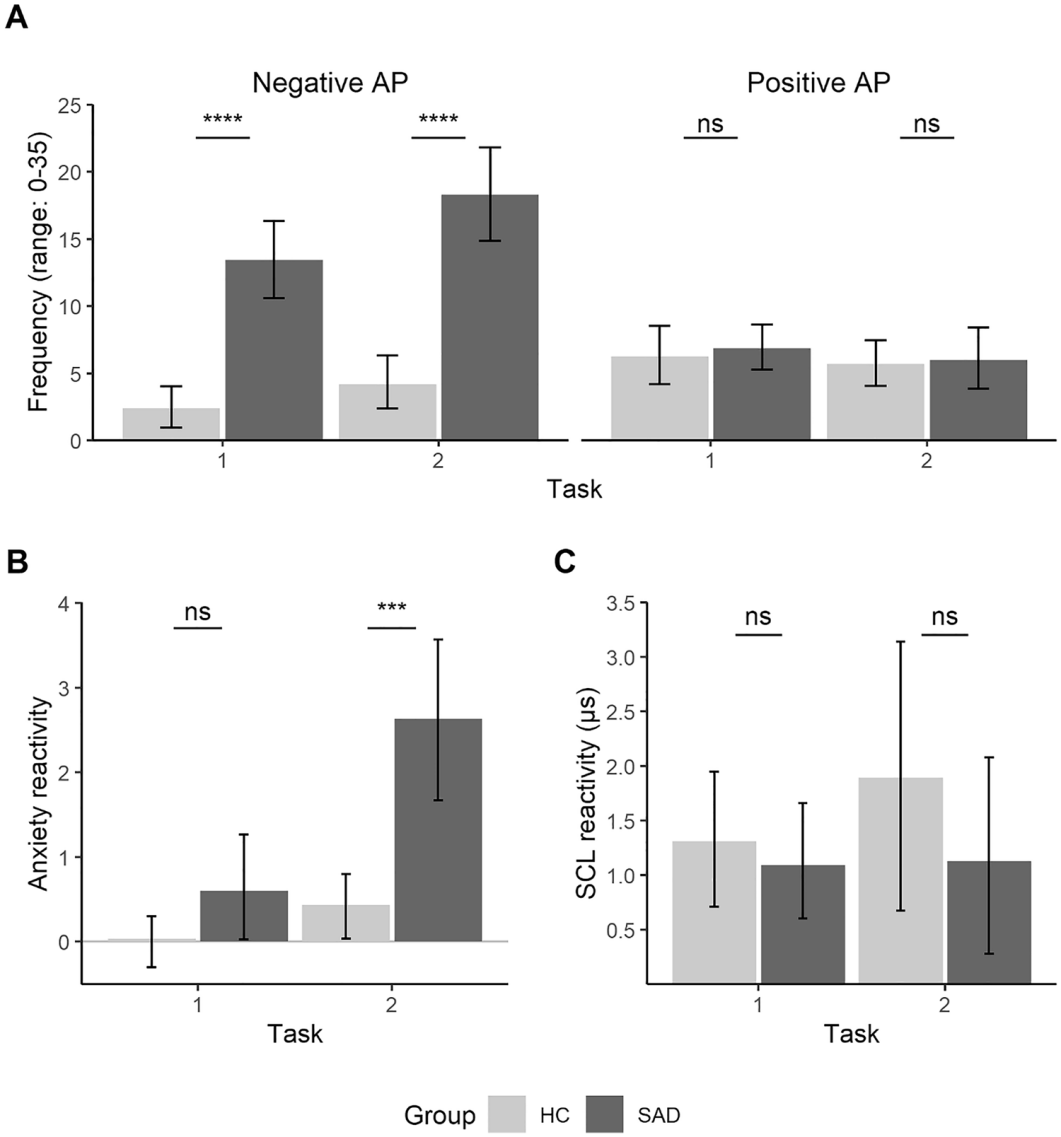
Frequency of Anticipatory Processing, Subjective Anxiety, and Skin Conductance Level in Anticipation of a Social Stress Task. *Note.* Panel **A:** Frequency of anticipatory processing (AP) as measured by the Thoughts Questionnaire for Children. Panel **B:** Subjective anxiety relative to baseline. Panel **C**: Skin conductance level (SCL) relative to baseline. HC = healthy control group; SAD = children with social anxiety disorder group. Error bars indicate 95% confidence intervals. ***p < .001. ****p < .0001

The analysis regarding Hypothesis 1a and b revealed significant main effects of Group, *F*(1, 58) = 34.88, *p* < 0.001, η_p_^2^ = 0.31, and Scale, *F*(1, 174) = 19.82, *p* < 0.001, η_p_^2^ = 0.10, as well as significant interactions of Group × Scale, *F*(1, 174) = 64.21, *p* < 0.001, η_p_^2^ = 0.27, and Scale × Task, *F*(1, 174) = 7.13, *p* = 0.008, η_p_^2^ = 0.04. All other included fixed effects were nonsignificant, all *F*s ≤ 2.98, *p*s ≥ 0.086. Follow-up analyses revealed that children with SAD reported significantly more negative anticipatory processing than children in the HC group in T1, *t*(202.7) = 6.45, *p* < 0.001, *d* = 1.63*,* 95% CI [1.04, 2.23] and T2, *t*(202.7) = 8.24, *p* < 0.001, *d* = 1.72, 95% CI [1.12, 2.33], whereas there was no significant difference between groups regarding positive anticipatory processing at T1 and T2, all *t* ≤ 0.37, *p* ≥ 0.713 (see Fig. 2). There were no significant differences between T1 and T2 in negative and positive anticipatory processing, all *t*s ≤ 1.19, *p*s ≥ 0.237.

#### Hypothesis 1c: Anticipatory Anxiety

The analysis regarding Hypothesis 1c revealed significant main effects of Group, *F*(1, 58) = 15.82, *p* < 0.001, η_p_^2^ = 0.21, and Task, *F*(1, 58) = 18.93, *p* < 0.001, η_p_^2^ = 0.25, and a significant interaction effect of Group × Task, *F*(1, 58) = 8.53, *p* = 0.005, η_p_^2^ = 0.13. Groups did not differ in subjective anxiety prior to the first task, *t*(110.9) = 1.27, *p* = 0.207, *d* = 0.40, 95% CI [-0.12, 0.93], but the group with SAD reported significantly higher subjective anxiety prior to the second task, *t*(110.9) = 4.93, *p* < 0.001, *d* = 1.10, 95% CI [0.54, 1.65].

#### Hypothesis 1d: Anticipatory SCL

The analysis regarding Hypothesis 1d did not reveal any significant main or interaction effects, all *F*s ≤ 1.35, *p*s ≥ 0.250.

#### Hypothesis 2: Relations Between Anticipatory Processing, Subjective Anxiety, and Performance Ratings

Stepwise regression models regarding Hypothesis 2a showed that negative anticipatory processing had significant incremental validity in the prediction of anxiety levels during both social stress tasks, when we controlled for age, trait social anxiety, and depression, *F*(1, 55) = 13.20, *p* < 0.001. Including positive anticipatory processing in the existing model did not improve the overall model fit significantly, *F*(1, 54) = 0.15, *p* = 0.702. In the final model including age, social anxiety, depression, and negative and positive anticipatory processing, only negative anticipatory processing significantly predicted anxiety levels during the social stress tasks (β = 0.499, *p* < 0.001). Regarding Hypothesis 2b, a similarly built stepwise regression model showed that negative anticipatory processing predicted subjective performance ratings assessed after the social stress task when we controlled for age, trait social anxiety, depression, and subjective anxiety during the social stress task, *F*(1, 54) = 14.66, *p* < 0.001. Including positive anticipatory processing in the existing model did not improve the overall model fit significantly, *F*(1, 53) = 1.62, *p* = 0.209. In the final model, age, subjective anxiety during the social stress tasks, and negative anticipatory processing were significant predictors of subjective performance ratings (all βs ≥ 0.217, all *p*s ≤ 0.031). The stepwise-built models as well as corresponding indices can be found in Table 2 and 3.

**Table 2:** Regression Coefficients (β) Explaining Variance in Subjective Anxiety Ratings During the Social Stress Task

Predictor	Step				
	I	II	III	IV	V
Age	-0.003	0.006	-0.014	0.023	0.023
SASC-R		0.698***	0.583***	0.186	0.189
CDI			0.162	0.165	0.169
Negative AP ^a^				0.505***	0.499***
Positive AP ^a^					0.034
Adjusted *R*^2^	-0.017	0.469	0.473	0.568	0.561
*R*^2^ change	-0.017	0.487	0.004	0.094	-0.007
*F* change	0.0004	54.19***	1.42	13.20***	0.15
AIC	294.72	256.63	257.13	246.23	248.06

Minimal tolerance = 0.27, maximal variance inflation factor = 3.68

*AIC* Akaike information criterion, *CDI* Children’s Depression Inventory, *SASC-R* Social Anxiety Scale for Children-Revised

^***^p < .001

^a^Negative and positive anticipatory processing (AP) measured by the Thoughts Questionnaire for Children

**Table 3:** Regression Coefficients (β) Explaining Variance in Subjective Performance Ratings Assessed After the Social Stress Task

Predictor	Step					
	I	II	III	IV	V	VI
Age	0.165	0.171	0.167	0.174	0.216*	0.217*
SASC-R		0.524***	0.498**	0.182	-0.167	-0.179
CDI			0.037	-0.050	-0.007	-0.021
State anxiety ^a^				0.540***	0.303*	0.312*
Negative AP ^b^					0.620***	0.635***
Positive AP ^b^						-0.115
Adjusted *R*^2^	0.010	0.277	0.265	0.408	0.526	0.531
*R*^2^ change	0.010	0.267	-0.012	0.143	0.118	0.005
*F* change	1.62	22.39***	0.05	14.56***	14.66***	1.62
AIC	179.65	161.77	163.71	151.62	139.22	139.41

Minimal tolerance = 0.265; maximal variance inflation factor = 3.77

*AIC* Akaike information criterion, *CDI* Children’s Depression Inventory, *SASC-R* Social Anxiety Scale for Children-Revised

^*^p < .05; **p < .01; ***p < .001

^a^Subjective anxiety during social stress task (relative to baseline)

^b^Negative and positive anticipatory processing (AP) measured by the Thoughts Questionnaire for Children

### Effects of Distraction on Post-event Processing, Subjective Anxiety, and SCL

#### Hypotheses 3 and 4a and b: Frequency of Negative and Positive Post-event Processing

**Fig. 3:**
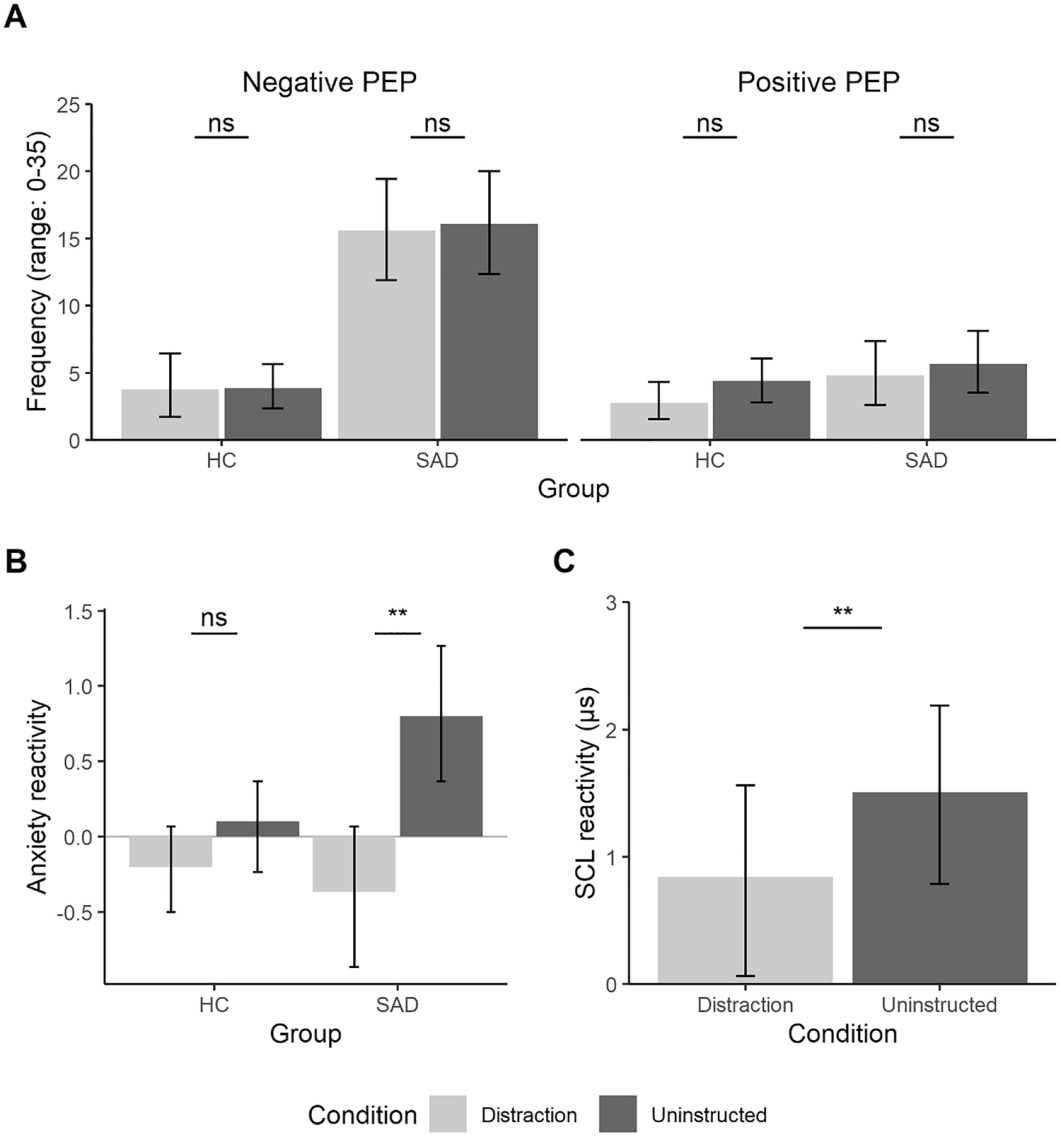
Effect of a Distraction Condition Compared to an Uninstructed Post-Event Processing Condition on Post-event Processing, Subjective Anxiety, and Skin Conductance Level*. Note.* Panel **A:** Frequency of post-event processing (PEP) as measured by the Thoughts Questionnaire for Children. Panel **B:** Subjective anxiety relative to baseline by group and condition. Panel **C:** Skin conductance level (SCL) relative to baseline by condition. HC = healthy control group; SAD = children with social anxiety disorder group. Error bars indicate 95% confidence intervals. **p < .01

The MLM regarding Hypotheses 3 and 4a and b showed significant main effects of Group, *F*(1, 56) = 24.71, *p* < 0.001, η_p_^2^ = 0.31, Scale, *F*(1, 168) = 60.26, *p* < 0.001, η_p_^2^ = 0.26, and Task, *F*(1, 168) = 7.72, *p* = 0.006, η_p_^2^ = 0.04, and significant interactions of Group × Scale, *F*(1, 168) = 55.16, *p* < 0.001, η_p_^2^ = 0.25, Scale × Task, *F*(1, 168) = 17.38, *p* < 0.001, η_p_^2^ = 0.08, and Group × Scale × Task, *F*(1, 168) = 5.74, *p* = 0.018, η_p_^2^ = 0.03. The main effect of Condition and all interaction effects including Condition were nonsignificant, all *F*s ≤ 1.67, *p*s ≥ 0.198. Irrespective of task, children with SAD showed significantly higher values of negative post-event processing than the HC group, T1: *t*(146.3) = 6.01, *p* < 0.001, *d* = 1.62, 95% CI [1.02, 2.12], T2: *t*(146.3) = 3.43, *p* < 0.001, *d* = 1.32, 95% CI [0.75, 1.89]. There were no significant differences in positive post-event processing in either task, all *t*s ≤ 1.19, *p*s ≥ 0.238. The group with SAD reported significantly less negative post-event processing in T2 compared to T1, *t*(146.3) = -3.04, *p* = 0.003, *d* = 0.66, 95% CI [0.42, 0.90], whereas there was no significant difference in negative post-event processing between the tasks in the HC group, *t*(146.3) = -0.45, *p* = 0.643, *d* = 0.43, 95% CI [0.22, 0.64]. Neither group differed significantly in positive post-event processing between T1 and T2, all *t*s ≤ 1.19, *p*s ≥ 0.237. The frequency of negative and positive post-event processing is shown in Fig. 3.

#### Hypothesis 4c: Effects of Distraction on Subjective Anxiety Levels

The analysis regarding Hypothesis 4c showed significant main effects of Condition, *F*(1, 56) = 20.81, *p* < 0.001, η_p_^2^ = 0.27, and Task, *F*(1, 56) = 13.94, *p* < 0.001, η_p_^2^ = 0.20, as well as significant interactions of Condition × Group, *F*(1, 56) = 7.27, *p* = 0.009, η_p_^2^ = 0.11, and Group × Task, *F*(1, 56) = 5.20, *p* = 0.026, η_p_^2^ = 0.09. All other main effects as well as interactions were nonsignificant, all *F*s ≤ 1.51, *p*s ≥ 0.224. The group with SAD reported significantly lower subjective anxiety levels in the distraction compared to the uninstructed post-event processing condition, *t*(103.3) = -3.32, *p* = 0.001, *d* = 0.78, 95% CI [0.25, 1.32], whereas there were no significant differences in anxiety between the two conditions in the HC group, *t*(103.3) = -1.75, *p* = 0.084, *d* = 0.46, 95% CI [0.09, 1.01]. The group with SAD reported significantly lower anxiety levels in T2 compared to T1, *t*(103.3) = -2.80, *p* = 0.006, *d* = 0.72, 95% CI [0.14, 1.30], whereas there was no difference in anxiety levels between T1 and T2 in the HC group, *t*(103.3) = -1.57, *p* = 0.119, *d* = 0.26, 95% CI [-0.13, 0.66].

#### Hypothesis 4d: Effects of Distraction on SCL

The analysis regarding Hypothesis 4d showed a significant main effect of Condition, *F*(1, 56) = 7.13, *p* = 0.010, η_p_^2^ = 0.11. SCL was significantly lower in the distraction condition than in the uninstructed rumination condition. All other included main and interaction effects were nonsignificant, all *F*s ≤ 1.54, *p*s ≥ 0.220.

## Discussion

Our aim was to investigate negative and positive anticipatory processing and its association with subjective anxiety, self-evaluations, and autonomic arousal in children with SAD compared to a HC group in an experimental laboratory social stress task. In addition, the impact of a cognitive distraction intervention implemented directly after the social stress task on negative and positive post-event processing, subjective anxiety, and autonomic arousal was tested.

### Anticipatory Processing

In line with our first hypothesis and cognitive models of SAD (Clark & Wells, 1995; Hofmann, 2007), children with SAD in our study reported significantly more negative anticipatory processing than children in the HC group. Importantly, these findings extend previous studies that used nonclinical community samples (Vassilopoulos et al., 2014). However, partly contradicting our first hypothesis, we found no evidence of elevated autonomic arousal during anticipation of the social stress tasks, and subjective anxiety in the group with SAD was elevated only in anticipation of the second social stressor, probably owing to stress sensitization in our clinical group (Asbrand, Heinrichs, et al., 2019a). In contrast, Vassilopoulos et al. (2014) demonstrated an association between an instructed anticipatory processing condition and elevated anxiety levels in a community sample aged 10 to 11 years. While Vassilopoulos et al. (2014) used an instructed negative anticipatory processing condition, we did not specifically instruct children to engage in anticipatory processing, to capture this process with higher ecological validity. It is likely that instructed anticipatory processing would lead to a significant amplification of ruminative processes and related qualities such as subjective anxiety, particularly in children whose cognitive processes are still developing and are not as distinctive as in adolescents or adults (Alfano et al., 2002).

In accordance with our second hypothesis, negative anticipatory processing statistically predicted elevated subjective anxiety experienced during the social stress tasks and lower subjective self-evaluations of performance. Importantly, negative anticipatory processing explained variance beyond trait and state anxiety, suggesting a unique contribution of this process to disorder maintenance in childhood SAD (Halldorsson & Creswell, 2017). In their cognitive model, Clark and Wells (1995) argued that anticipatory processing may activate a complex dysfunctional processing pattern consisting of negative interpersonal self-beliefs and self-directed attention. This results in a negative cognitive-emotional processing of the feared social situation, for example, negatively biased self-evaluations and heightened subjective anxiety during social stress, as found in our study. The consideration of negative anticipatory processing as part of a complex maintenance process seems to be especially important in light of the small to medium amount of explained variance found in our study.

Positive anticipatory processing was comparable between the experimental groups and did not predict anxiety levels or subjective performance ratings in the social stress tasks. This indicates that SAD in children may be characterized more by elevated levels of negative anticipatory processing than by an absence of positive thoughts. This corresponds to the notion that a higher frequency of negative thoughts, as opposed to the presence of positive thoughts, is associated with psychopathology (Kendall & Chansky, 1991).

### Post-event Processing and Effects of Cognitive Distraction

In accordance with our third hypothesis and previous research in socially anxious children (Asbrand, Schmitz, et al., 2019b; Schmitz et al., 2010), children with SAD reported more negative post-event processing than children in the HC group. Thus, children with SAD experienced more dysfunctional cognitions regarding their performance, the observers, and their appearance during the social stress task. Since previous research has shown that post-event processing is highly related to the maintenance of negative self-perceptions and biased self-evaluations in affected children (Miers et al., 2014; Schmitz et al., 2011), effective psychotherapeutic interventions to target and alter negative post-event processing are needed (Asbrand, Schmitz, et al., 2019b). In this context, our study is to our knowledge the first to investigate the effects of a cognitive distraction intervention on post-event processing in childhood SAD. Partly confirming our fourth hypothesis, distraction was associated with reduced anxiety levels in the group with SAD and reduced autonomic arousal in all participants (e.g., Wong & Moulds, 2009). However, in contradiction to our expectations and results from previous studies with highly socially anxious undergraduates (Blackie & Kocovski, 2016; Kocovski et al., 2011), the implemented distraction condition did not affect the frequency of negative or positive post-event processing. Several explanations are conceivable for these differences. First, most of the previous studies on the effects of distraction on post-event processing used an instructed post-event processing condition, thus specifically amplifying negative cognitions probably beyond their natural occurrence (e.g., Blackie & Kocovski, 2016). By contrast, our study used an uninstructed post-event processing condition, which may have resulted in a higher ecological validity but probably also smaller effects of distraction than when compared to instructed post-event processing. Rowa et al. (2014) reported results similar to ours, that is, a positive influence of a distraction condition compared to a post-event processing condition on anxiety levels, but no reduction of negative post-event processing in an adult SAD sample. They suggested that a distraction task may provide anxiolytic effects even alongside the presence of naturally occurring negative post-event processing. A similar process may have occurred in our sample, further indicating that negative post-event processing may be associated with stable negative self-schemata that may be reduced only with more extensive cognitive interventions. Previous developmental research has shown that children experience negative emotions and related cognitions more intensely (e.g., no mixed states of both positive and negative cognitions; Alfano et al., 2002). As a consequence, children have more difficulties profiting from cognitive interventions such as distraction due to an intense subjective experience of negative cognitions and emotions. Children may consequently need training and instructions beyond the relatively mild form of a single-session distraction condition used in our study (Volkaert et al., 2020).

### Limitations, Future Directions, and Clinical Implications

There are several limitations to the current study. First, it is unclear to what extent our participants were distracted by the tablet game. We chose not to ask children about the extent of distraction because a major goal of our study was to measure anticipatory and post-event processing without strong reactivity and social desirability effects. Still, given the reduced levels of subjective anxiety and SCL in the distraction condition, we assume participants were overall able to successfully engage in distraction. Second, although we found positive short-term effects of distraction, further research is needed to study possible detrimental long-term effects. This is particularly important as distraction may be conceptualized as covert avoidance or safety behavior in SAD depending on its specific implementation (Clark & Wells, 1995). Third, although our sample size was sufficient according to an a priori power analysis, future studies may want to replicate our results in larger or more diverse samples by including children covering a larger age range and by comparing children with SAD to other clinical groups. Fourth, the study’s cross-sectional design prevents the determination of causal effects and our results may not be directly transferable to social interactions, as these are often more ambiguous and require a different skill set than social performance situations (Voncken & Bögels, 2008).

Future studies are needed to continue to examine the predictors and consequences of negative anticipatory and post-event processing in children with SAD. Both ruminative processes are likely part of a complex SAD-maintaining network (Hirsch et al., 2006; Wong, 2016); hence, experimental research focusing on several key aspects of cognitive models and their interconnections, for example, self-focused attention and its associations with rumination, is essential. Continuing research efforts are further needed to refine interventions to target anticipatory and post-event processing and to integrate them in a comprehensive intervention concept, for example, SAD-specific CBT (Leigh & Clark, 2018). Future studies should therefore compare the effectiveness of different therapeutic strategies (e.g., cognitive reappraisal; Helbig-Lang et al., 2015; Shikatani et al., 2014), methodological approaches, and intervention intensities in altering ruminative processes in childhood SAD.

In conclusion, the present findings suggest that SAD in children is associated with elevated levels of negative anticipatory and post-event processing, which are thought to be maintenance factors of the disorder in cognitive models of adult SAD (Clark & Wells, 1995). Negative anticipatory processing was further associated with elevated anxiety and reduced subjective performance ratings, suggesting possible detrimental effects of said process. The implemented distraction condition did not reduce negative post-event rumination in our sample but had a positive effect on emotional and physical poststress arousal.
